# Quality Assessment of Protein Docking Models Based on Graph Neural Network

**DOI:** 10.3389/fbinf.2021.693211

**Published:** 2021-08-12

**Authors:** Ye Han, Fei He, Yongbing Chen, Wenyuan Qin, Helong Yu, Dong Xu

**Affiliations:** ^1^ School of Information Technology, Jilin Agricultural University, Changchun, China; ^2^ Department of Electrical Engineering and Computer Science, Christopher S. Bond Life Sciences Center, University of Missouri, Columbia, MO, United States; ^3^ School of Information Science and Technology, Northeast Normal University, Changchun, China

**Keywords:** protein docking, quality assessment, graph neural network, graph attention network, ensemble learning

## Abstract

Protein docking provides a structural basis for the design of drugs and vaccines. Among the processes of protein docking, quality assessment (QA) is utilized to pick near-native models from numerous protein docking candidate conformations, and it directly determines the final docking results. Although extensive efforts have been made to improve QA accuracy, it is still the bottleneck of current protein docking systems. In this paper, we presented a Deep Graph Attention Neural Network (DGANN) to evaluate and rank protein docking candidate models. DGANN learns inter-residue physio-chemical properties and structural fitness across the two protein monomers in a docking model and generates their probabilities of near-native models. On the ZDOCK decoy benchmark, our DGANN outperformed the ranking provided by ZDOCK in terms of ranking good models into the top selections. Furthermore, we conducted comparative experiments on an independent testing dataset, and the results also demonstrated the superiority and generalization of our proposed method.

## Introduction

A major way for proteins to perform their functions is through interacting with other proteins and producing protein complexes (Tuncbag et al., 2011). Protein-protein interactions play important roles in key activities and pathways in cells (Midic et al., 2009; Tu et al., 2015). The three-dimensional (3D) structure of a protein complex offers a deeper insight into the molecular mechanism of its biological function. Especially the interfaces at protein complexes are often considered as prospective targets to block protein interactions in drug discovery (Veselovsky et al., 2010). Several experimental techniques can be used to obtain a 3D structure of protein complexes, such as X-ray crystallography, NMR and cryo-EM (Topf et al., 2008). However, due to the cost and labor required in these experimental techniques, it is often more feasible and efficient to model the 3D structure of protein complex *in silico* (Choi et al., 2009; Abdel et al., 2014). Computational protein-protein docking methods enable prediction of preferred binding conformations (Lengauer and Rarey, 1996), which may provide a structural basis for facilitating the development of drugs to successfully disrupt protein-protein interactions (Bienstock, 2012). Since antibody as a particular category of proteins produced by the immune system is capable of binding with high specificity to an antigen, protein docking tools are also adopted to generate accurate antigen-antibody complexes for evaluating the diversity of polyclonal responses in vaccine development (Rosalba et al., 2017; Weitzner et al., 2017).

There are two major approaches for the mainstream of protein docking systems. The first approach obtains docking results from experimental binding conformations of similar protein pairs, which is known as template-based methods (Tuncbag et al., 2011; Ivan et al., 2015). However, the limited available templates may affect the success of docking results. The more general approach is template-free docking to directly predict the 3D structure of protein complex prediction from two individual protein structures (Katchalski-Katzir et al., 1992; Gabb et al., 1997; Vakser, 1997). Generally, such *ab initio* methods sample all possible binding candidates first and then evaluate them with a quality assessment (QA) function (Hurwitz et al., 2016; Sekhar, 2016). The sampling step carefully searches over the rotational and translational degrees of freedom, and generates a large number of candidate docking models where the two proteins contact each other without significant steric clashes (Bernauer et al., 2007). And then the QA is launched to pick the native-like models from all candidates. Basically, with an exhaustive sampling approach, the near-native models are most likely covered in the numerous candidates (Lemmon and Meiler, 2013). Thus, the accuracy of QA directly determines the final docking results; however, it is still the bottleneck of current protein docking systems.

The classical QA algorithms can be roughly classified into three categories: physical energy-based (Dominguez et al., 2003; Brian et al., 2013; Mieczyslaw et al., 2013; Pierce et al., 2014), statistical potential-based (Gidon et al., 1999; Zhou and Zhou, 2002; Pons et al., 2011) and machine learning-based methods (Fink et al., 2011). The first two methods highly depend on the descriptors of geometric complementarity and physico-chemical features, such as van der Waals interactions, electrostatic effects, solvation, and so on. However, physical energies are often very sensitive to small variations and statistical potentials may not be sensitive enough to obtain good discerning power so that they have major limitations (Kitchen et al., 2004). Machine learning-based approaches were proposed to combine various features in a data-driven fashion for better generalization (Fink et al., 2011). Especially, since deep learning as the cutting-edge machine learning technology shows powerful predictive ability, a 3D convolutional neural network (3DCNN) was applied on the 3D structures of protein-protein interfaces to determine if docking models were native-like structures (Wang et al., 2021). This 3DCNN method showed a capacity to learn local spatial geometric properties of residues/atoms located at the protein-protein interface with a fixed kernel size but truncated the residue/atom interactions outside the kernels. Furthermore, a graph neural network (GNN) was applied at the atomic level to rank docking candidates (Wang et al., 2021). Although extensive efforts have been made in QA, a highly accurate and generalized QA scheme is yet to achieve.

To further explore the potential of deep learning technology in QA, we designed a deep graph neural network, named Deep Graph Attention Neural Network (DGANN), for docking model QA. Docking sampling candidates can be naturally represented as graphs according to their 3D structures, in which residues are treated as nodes and residue-residue contacts as edges so that the QA process can be formulated as a graph classification problem. GNN as a graph-oriented deep learning architecture, can learn node latent representations across the global topology of a graph. Such an advantage provides an adaptive way to generate deep residue (node) representations considering neighboring residue interactions, energetic contributions and local graph topology. So that GNN can distinguish near-native models by examining these inter-residue representations at protein-protein interfaces.

## Results

### Pipeline of Our Method

**FIGURE 1:**
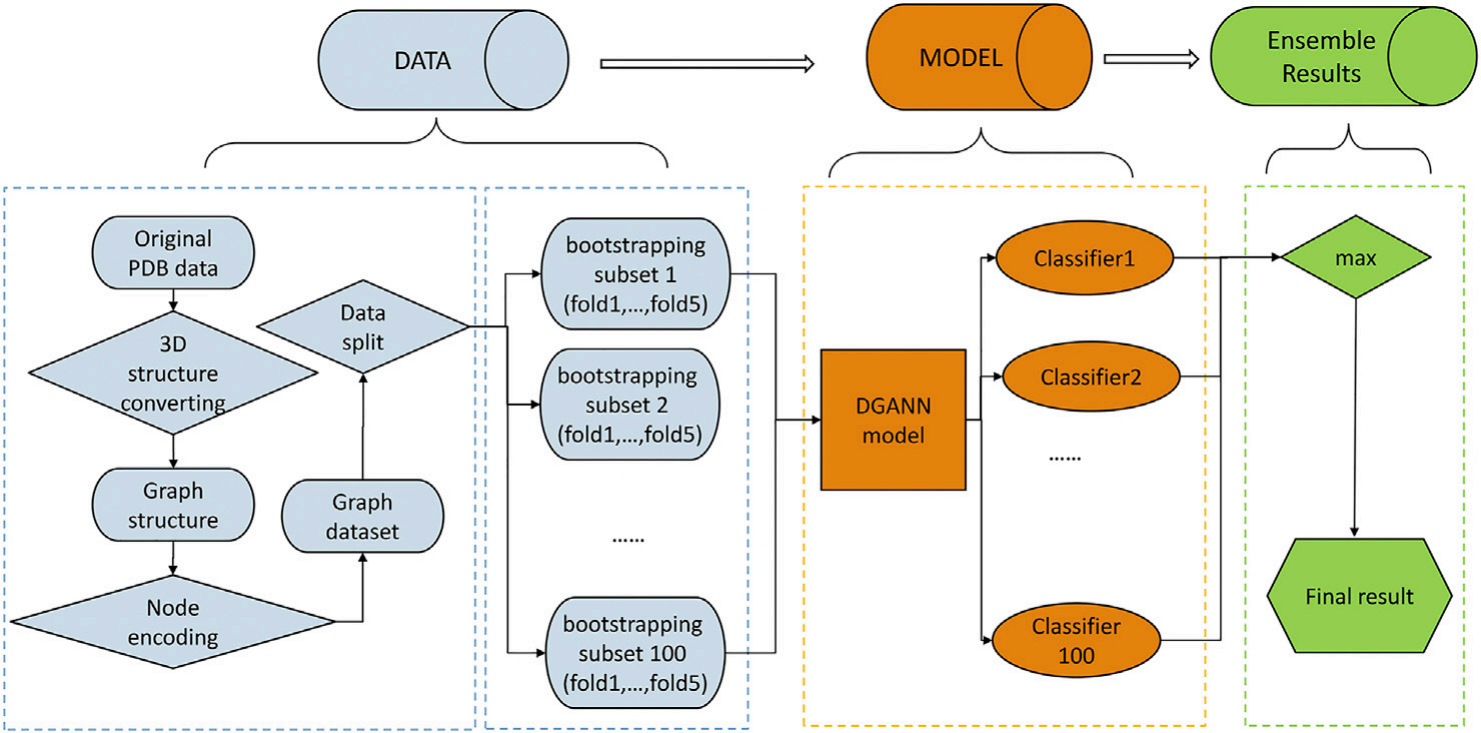
Flowchart of our method. The pipeline of our method includes three stages: 1) Data preprocessing stage (in blue): The PDB files of candidate docking models were transformed into graph structures, where each node is a residue, and each edge connects the two residues carrying any atoms within 5 Å interatomic distance. Then, we encoded each residue (node) by its physico-chemical properties and conservation profiles (detailed in Method C). All candidate docking models were labeled as positive or negative samples according to the Critical Assessment of PRedicted Interactions (CAPRI) criteria (defined in Methods E). We split all collected protein complexes into training and test sets for 5-fold cross-validation. 2) DGANN modeling stage (in orange): Aiming to address the imbalanced issue in QA, we bootstrapped 100 balanced training sub-sets to train our proposed DGANN to get 100 classifiers. 3) Ensemble learning stage (in green): An ensemble learning strategy was employed to integrate the outputs from the 100 classifiers. When assessing a protein docking model, we applied all classifiers to predict its quality scores and took their maximum score as the final prediction.

We formulated the protein docking model QA process as a binary classification problem, which takes a 3D structure of the candidate docking model as the input and outputs its probability of a near-native model. The framework of our proposed method is shown in Figure 1. Based on our formulation, any GNN-based classification method can be applied to conduct protein docking model QA.

**FIGURE 2:**
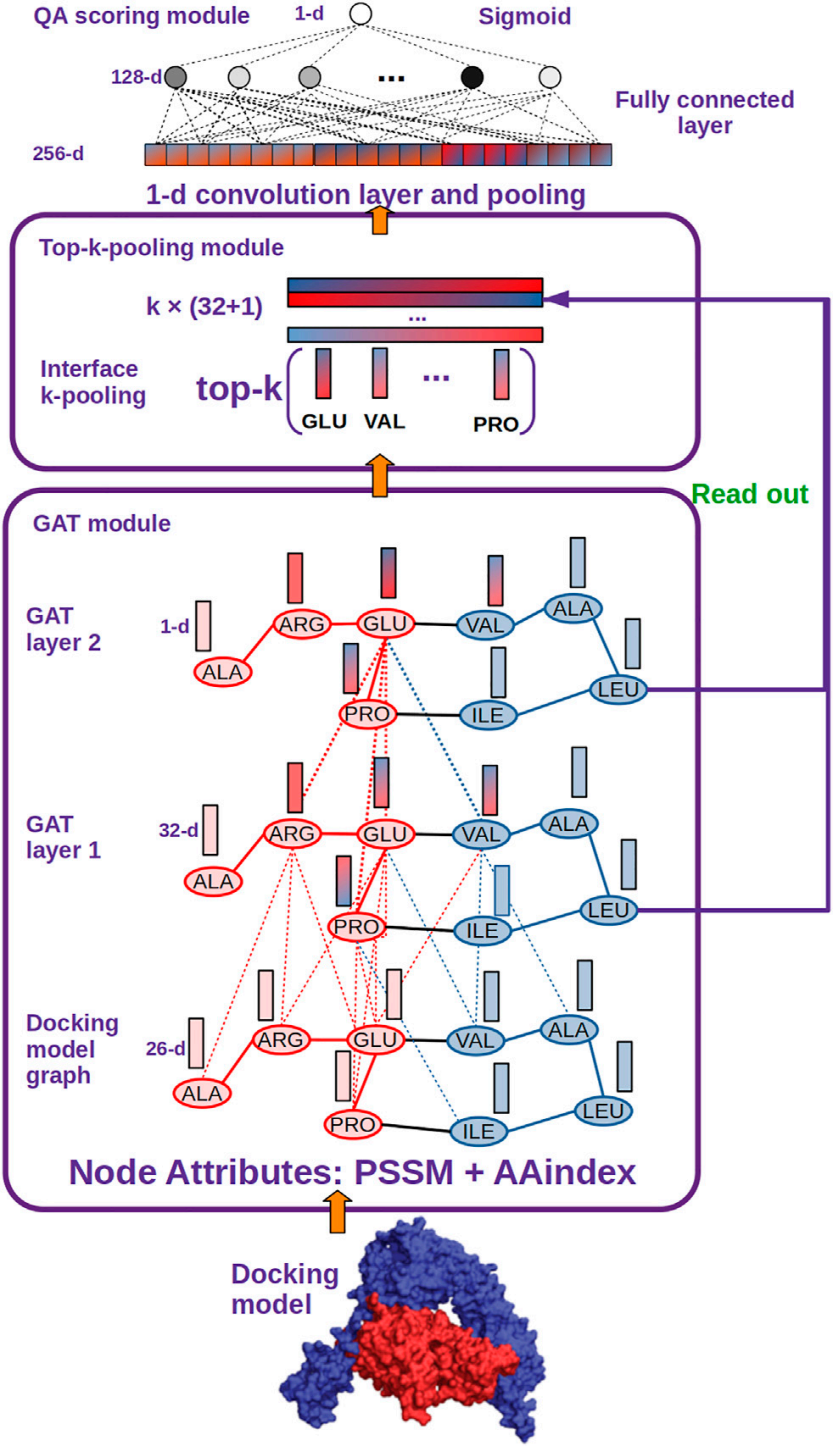
DGANN architecture. DGANN consists of three modules: 1) GAT module: Docking models are first represented as graphs (blue nodes denote the residues from the protein in blue, and red nodes represent the residues from the protein in red), where the nodes have 26-dimensional attributes. Then, two stacked GAT layers are designed to model neighboring residue interactions and local structural information. For instance, the node embedding of GLU at the second GAT layer comes from the attention-weighted aggregation of its neighbors ARG, PRO and VAL, whose embeddings are also aggregated by their neighbors at the first GAT layer. Through these processes, residue interactions from internal and external residues are modeled in each node embedding. Furthermore, at the first GAT layer, each aggregated node embedding is mapped to 32 dimensions, while at the second GAT layer the aggregated 32-dimensional node embeddings are mapped to a scalar for each node, which is deemed as the importance of the node over the whole graph. 2) Top-k-pooling module: To obtain a fixed size of graph-level representations, all residues at protein-protein interfaces are sorted by their importance, and the top k nodes are kept. And the two GAT outputs of the selected k nodes are concatenated to form graph-level representations of protein-protein interfaces. 3) QA scoring module: The graph representation of a docking model is fed into a 1-D convolutional layer and a fully connected layer to generate a flattened feature vector. Finally, a sigmoid function is applied to compute its probability of a native-like model.

In this paper, we proposed DGANN to implement it, which was adapted from Deep Graph Convolutional Neural Network (DGCNN) (Zhang et al., 2018) as shown in Figure 2. In DGANN, we replaced the Graph Convolutional (GCN) layers in DGCNN with Graph Attention (GAT) layers. This type of layers can learn node embeddings by aggregating their neighbors’ features using self-attention, which specifies different weights to different neighboring nodes according to their node attributes (Velikovi et al., 2017). Compared with directly summing neighboring node features as aggregation in GCN, node embeddings by the GAT-style aggregation implicitly illustrate unique residue interactions and local topology information from different neighboring residues.

In practice, we build the graph on the entire docking model with learn-deep representations of all residues by stacking GAT layers over the whole graph. By doing this, embedding of each node can model its interactions with surrounding residues. Especially the residues at the edge of the docking interface can aggregate information not only from neighbors inside the interface but also other residues outside the interface. Based on these residue (node) embeddings, we further generated protein-protein interface representations and conducted QA scoring in DGANN as Figure 2 described. Since few docking candidates from the sampling stage were native-like models, the distribution of positive and negative samples was extremely imbalanced. We employed ensemble learning to overcome such an imbalanced problem, which can generate stable and unbiased predictions by combining multiple DGANN models from bootstrapping balanced datasets.

### Comparisons With ZDOCK

For fair comparisons, we benchmarked on the docking models from ZDOCK (detailed in *Datasets* Section), since ZDOCK can generate all docking models and their QA results using the ZDOCK scoring function. Table 1 lists the enrichment factor (EF, defined in *Evaluation Metrics* Section) of good models at different top ranks from our ensemble DGANN results and ZDOCK results. In this experiment, we tried the mean and maximum scores from all meta-classifiers as ensemble results and checked their EFs at 0.01, 0.1, 0.5, 1, 5 and 10% top-scores as shown in Table 1. It can be found that our ensemble results achieved better EFs than ZDOCK at various checkpoints. Especially at the stringent checkpoints including EF at 0.01, 0.1, 0.5 and 1%, our ensemble DGANN respectively showed 33.5, 33.4, 27.5 and 33.5% higher enrichment than ZDOCK, which demonstrated our proposed DGANN had a greater capacity to find true positives throughout various scales of docking candidates. Such overall outperformance also suggested DGANN offered a better way to model inter-residue interactions and physico-chemical contributions following the topology of protein docking models, and generate informative representations for QA scoring. In addition, our ensemble strategy also indicated a capacity for enhancing near-native models from a huge amount of low-quality docking sampling candidates. Meanwhile, it is also observed that compared with taking the mean scores as ensemble results, the maximum scores worked much better at most checkpoints. A possible reason is that maximum scores from all meta-classifiers imply the highest probabilities to native-like models the testing model can achieve, which is beneficial to highlight potential positives from such extremely imbalanced data.

**TABLE 1:** EF comparisons between our ensemble DGANN and ZDOCK.

Methods	EF_0.01%_ top1	EF_0.1%_ top4	EF_0.5%_ top18	EF_1%_ top36	EF_5%_ top180	EF_10%_ top360
ZDOCK	12.11	12.79	8.66	6.48	3.75	2.81
Ensemble-mean	7.35	9.74	9.76	8.25	**4.78**	**3.61**
Ensemble-max	**16.17**	**17.06**	**11.05**	**8.65**	4.61	3.43

The bold values represent the best result at each column.

**FIGURE 3:**
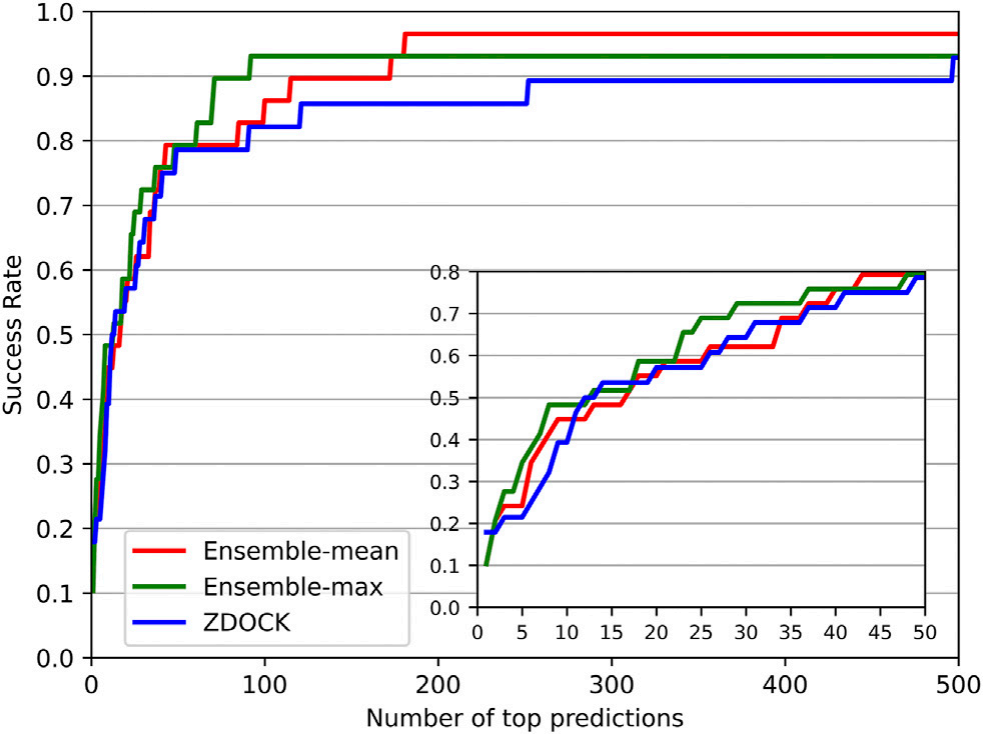
Success rates for DGANN and ZDOCK at the whole range and zoomed-in at top 50 ranks.

We also investigated the success rate (defined in *Evaluation Metrics* Section) at different top ranks of DGANN and ZDOCK. Figure 3 shows that DGANN and ZDOCK achieved similar AHCs at top ranks but displayed different trends afterward. This is because EF introduced EF_max_ to remove the biases caused by different imbalanced degrees among different protein complexes, while the success rate only reported an overall ability to hit positives (Wang et al., 2021). Due to the stronger enrichment ability to difficult cases, DGANN gained superior EFs and comparable success rates to ZDOCK at top ranks. With the growth of ranks, both our ensemble results outperformed ZDOCK on the success rate. Moreover, between two ensemble modes, the mean score mode obtained slightly better success rates than the maximum score mode at most top ranks. This indicates that the max ensemble mode tends to well handle hard cases, and the mean ensemble mode is better suited to work on general cases.

### Hyper-Parameter Optimization

#### n-hop Neighboring Aggregation Optimization

**FIGURE 4:**
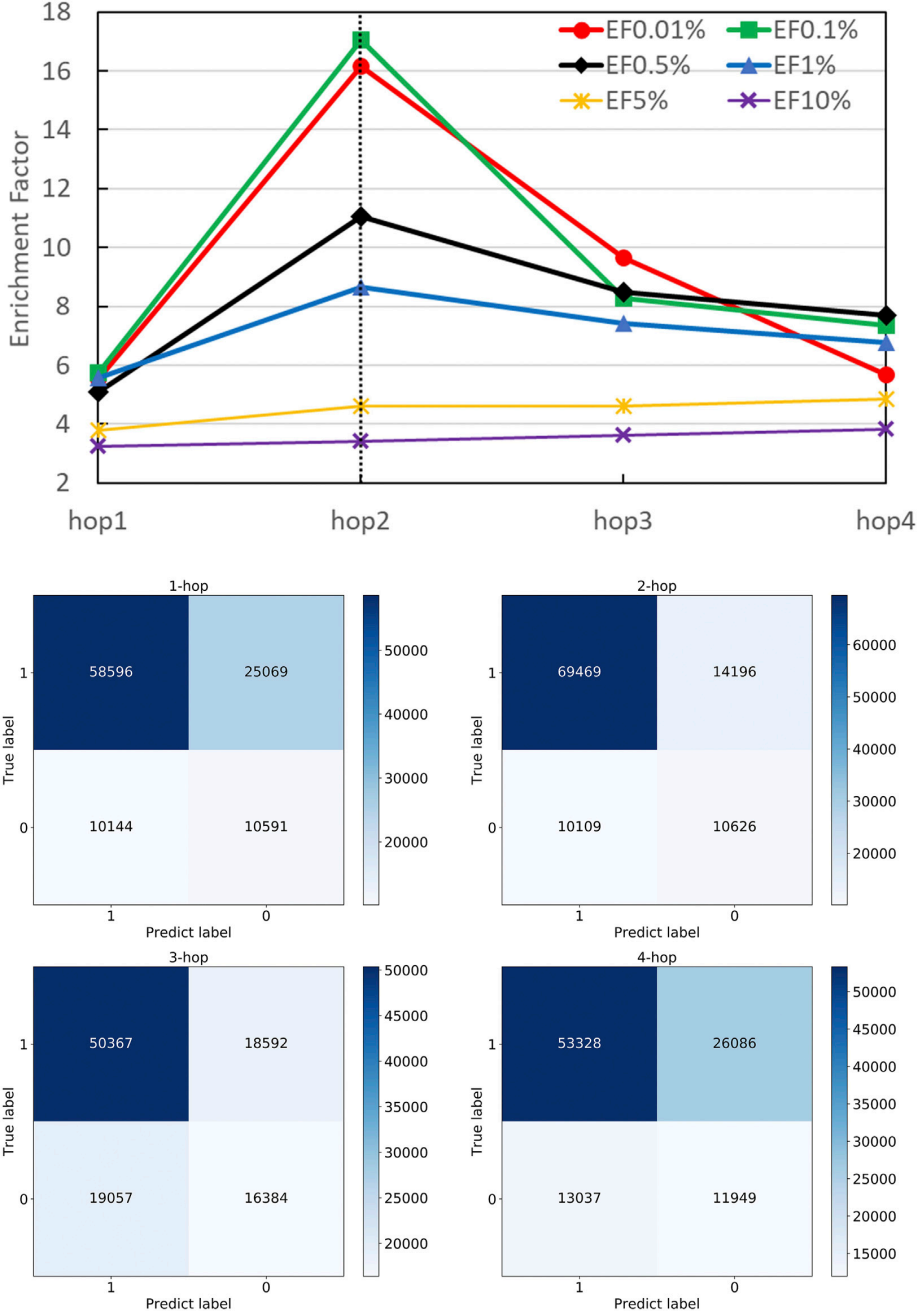
Performance on different numbers of hops. The number of hops means the number of GAT layers stacking in DGANN, which determines the longest distance neighboring aggregation in node embedding. We examined hop-1 to hop-4 setting and found hop-2 achieved the best EFs at different checkpoints.

A strength of DGANN is utilizing the GAT layer to aggregate neighboring residue properties for capturing residue interactions and structural information. The number *n* of stacking GAT layers leads to aggregate n-hop neighboring residues. For example, 1-hop represents the nearest neighbors and 2-hop also includes the next-nearest neighbors. We stacked different numbers of GAT layers and collected their confusion matrices and EF performance as shown in Figure 4. Particularly, in the 1-hop experiment, we assigned a single GAT layer to provide node importance coefficients for top-k-pooling but directly read out the input node attributes of pooled *k* nodes to the QA scoring module for prediction. Thus, DGANN without neighboring aggregation would degenerate to a classical deep neural network.

Figure 4 shows that DGANN with 2-hop aggregation achieved more true positives and true negatives, as well as better EF at 0.01, 0.1, 0.5 and 1% than those of 1-hop setting. The performance exhibits the superiority of DGANN in characterizing protein docking models compared with classical deep learning architectures.

It indicates that the neighboring aggregation operation enables us to learn residue interactions and topology information at contact interfaces to predict native-like models. Meanwhile, more than 2-hop architectures did not bring further improvement in EF. These results suggest considering too many hops in residue (node) embeddings may introduce redundant and noisy residue interactions and damage their discerning power.

### Different k Optimization

In DGANN, the top-k-pooling module picks the best *k* residue (node) embeddings at the interface region to define the fixed size of graph-level representations for further QA scoring. Therefore, it is necessary to select a reasonable *k* to keep generalization and prevent information loss across all protein complexes. For the cases with fewer than *k* nodes, we padded the placeholders 0 to fix the output of top-k-pooling.

**FIGURE 5:**
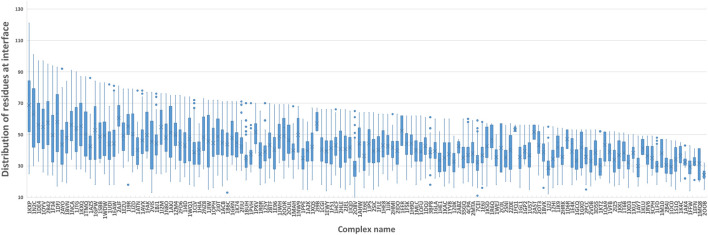
Boxplot of residues at the protein-protein interface. We computed the number of residues at the interface of each training sample and grouped them by protein complex names. The boxplot is to visualize the distribution of residues at interfaces of all docking models for each protein complex.

Figure 5 shows the number of residues at the contact interface from our training protein complexes. Then, we tried their mean 40, maximum 120, and other values such as 80 and 100 as the *k,* to show its influence on QA results. Another option to squeeze node-level features with various shapes to graph-level features with a fixed shape is the pooling operation. Here, we also attempted both global max pooling and global mean pooling over all nodes at the interface.

**FIGURE 6:**
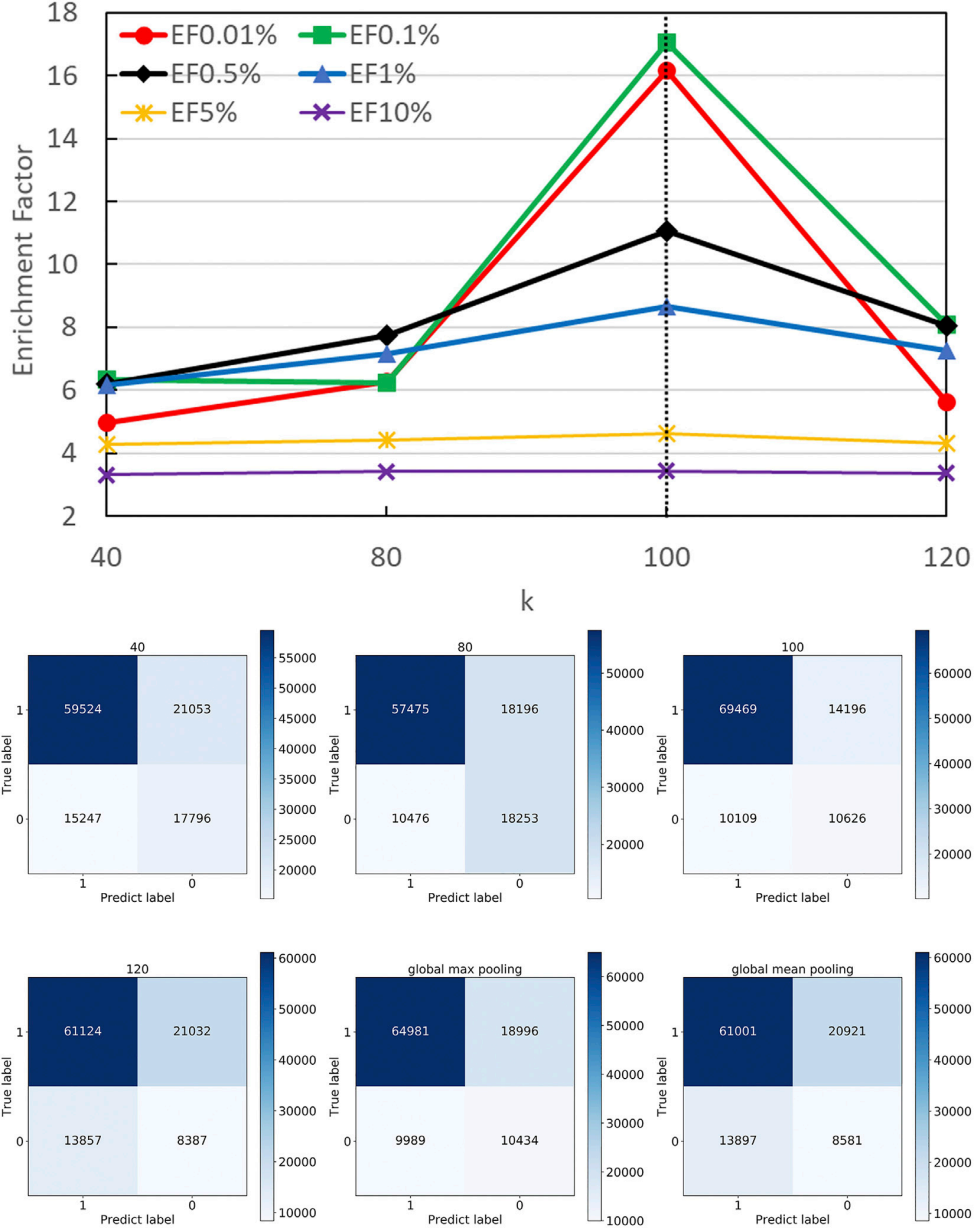
Performance on different pooling *k*. The hyper-parameter *k* determines the number of remaining nodes in modeling graph-level representations. We attempted different *k* values according to the distribution of residues at protein interfaces along with two types of global poolings and found 100 is roughly the best option.

The confusion matrices and EFs of different *k* are shown in Figure 6. When *k* equals 100, the top-k-pooling strategy slightly outperformed other k options and two global pooling approaches in terms of true positives, true negatives and EFs. According to Figure 5, only a few protein complexes carried more than 100 residues at the contact interface. The optimal *k* value implies that almost all the residues at the interface provide informative features and contribute to the selection of near-native models. Between the two global pooling methods, mean-pooling took the total information from all interfacial residues while max-pooling kept significant signals from interfacial residues. The top-k-pooling with an optimal k can make a good balance, so that it achieved relatively better performance.

#### Effect of Similar Targets

**FIGURE 7:**
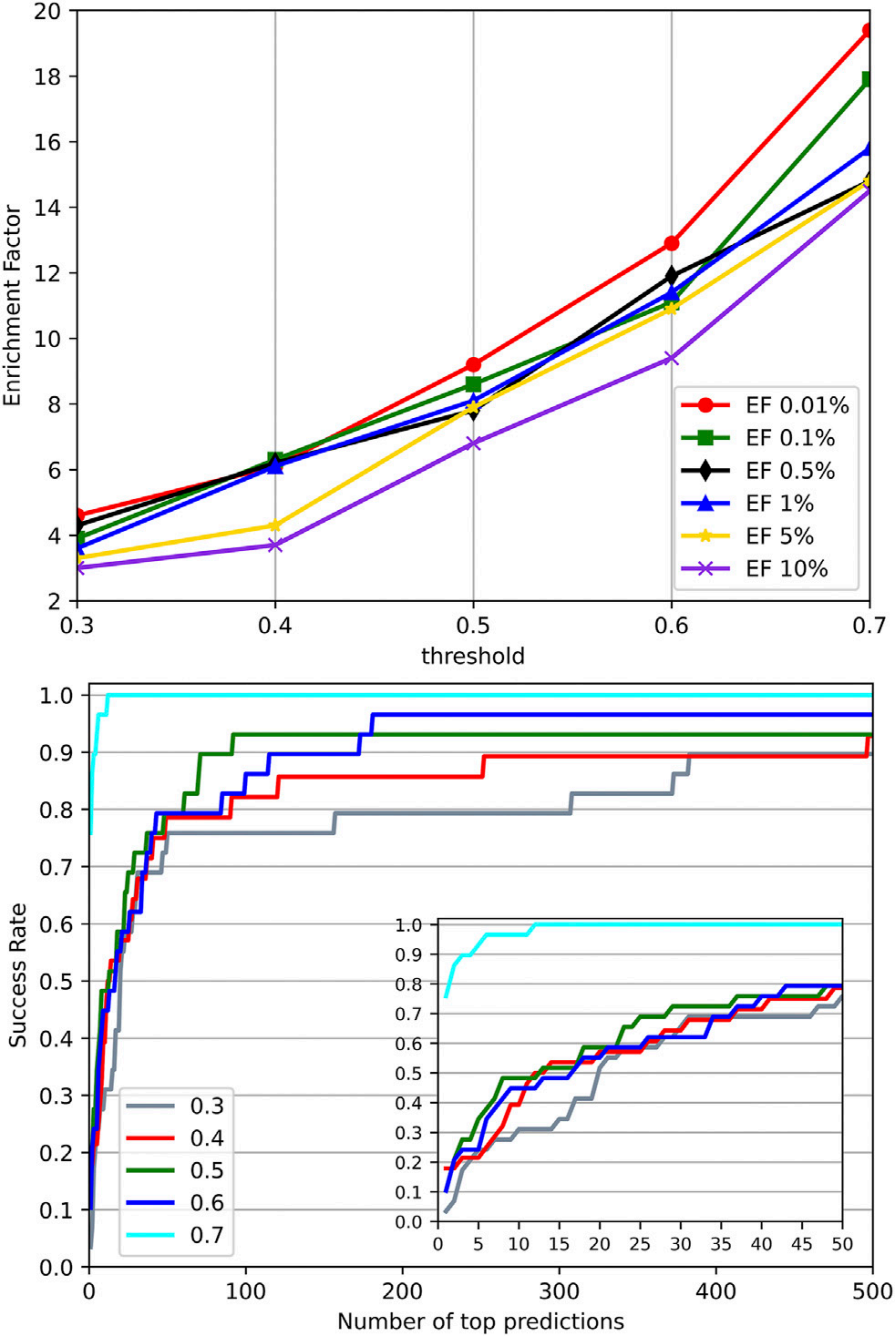
Performance on different identity thresholds. We explored different identity thresholds in CD-HIT clustering and resulted in several groups of the training set and test set. The result showed that the common adopted threshold 0.4 may be stringent enough to control the similarity between the training set and test set.

Significant similarity between the training data and the test data may cause performance overestimation for a machine learning model due to overfitting. To investigate the effect of similar targets between the training set and test set on the performance, we went through different identity thresholds in CD-HIT (Ying et al., 2010) clustering, which resulted in several groups of training sets and test sets. A series of experiments on these groups of training sets and test sets were conducted. Their EFs and success rates were plotted in Figure 7. It can be observed that with the increased identities, the groups with higher similarity between the training set and test set performed much better than the groups with low similarity. The results suggest that high similarity between targets of the training set and the test set may bring an overestimation to the method evaluation. However, we employed a relatively stringent threshold 0.4 in splitting datasets to provide reasonable experimental control. In the experiment using an identity threshold of 0.3, the EFs and success rates were only dropped slightly, which demonstrates the commonly-adopted threshold 0.4 may be stringent enough to control the similarity between the training set and the test set.

### Comparisons With Existing Tools

DOVE (Wang et al., 2019) and iScore (Geng et al., 2019) are the latest accessible tools for ranking docking models of protein–protein complexes. DOVE trained eight sub-models using various features named Atom20, Atom40, Goap, iTscore, and their combinations. Atom20 and Atom40 mean using the thresholds of atoms to locate protein interfaces. And the Goap and iTscore are the employed tools in protein feature extraction. In their paper, DOVE-Atom40 outperformed other sub-models. And iScore performed consistently better than the SwarmDock, pyDock, Zdock in their paper. In addition, ZDOCK and HADDOCK were among the top popular docking tools, and provided not only docking models but also their QA scores. Therefore, we compared our DGANN with DOVE-Atom40 and iScore on an independent test set. The test samples were collected from Protein-Protein Docking Benchmark 5.5, which was updated in 2020 when DOVE and iScore were published. The newly collected 97 target complexes also do not appear in our modeling and previous evaluation.

**FIGURE 8:**
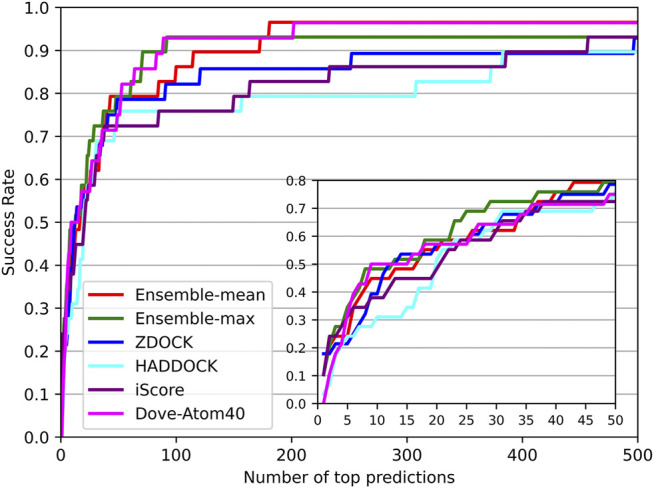
Decoy selection performance on the independent test set.

The success rates on the independent dataset were shown in Figure 8. Among the five tools, DGANN showed outstanding performance on the independent dataset. At the top 5 ranks, DOVE-Atom40 achieved a success rate of 0.3, outperforming the other four tools, while DGANN received 0.36. We noticed that both DGANN and DOVE-Atom40 also employed physico-chemical properties. While our method also benefits from physico-chemical properties, the graph neural network can implicitly embed the topology of protein-protein structural information. We have also computed the enrichment factor (EF) as the measurement of docking model selection.

**FIGURE 9:**
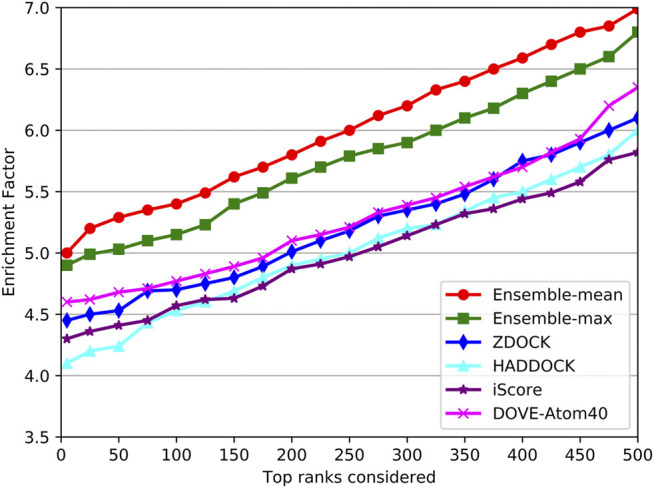
Comparison of the EF on the independent test set.

As shown in Figure 9, the trends of EFs on the three tools were similar to success rates. The EF curve of DGANN was above those of DOVE-Atom40 and iScore. We believed the reason is the message passing operator of DGANN can simulate the interaction between amino acids and mimic the micro-environment for better generating the representations of docking models. Nevertheless, the overall performance of our method on benchmark 5.5 cannot compare to those on benchmark 4.0, which reflected the more complicated cases of protein complexes were collected in benchmark 5.5. That required further improved docking tools and better QA approaches to provide accurate docking models.

## Materials and Methods

### Datasets

In our work, we evaluated DGANN on a ZDOCK decoy set, which provided all docking models and their QA racking results. The dataset covered the docking sampling results from ZDOCK3.0.2 on 176 protein complexes from protein docking benchmark 4.0 (Hwang et al., 2010), each of which included 3,600 sampling candidate models. To avoid overestimation in our evaluation, we applied CD-HIT (Ying et al., 2010) to cluster the targets with 40% identity cutoff, which was a common threshold in the literature. We combined its clustering results into three groups to form our training set, validation set and test set. Such combination carried out 5 times resulting in 5-fold datasets for cross-validation. The identity threshold ensures that relatively low similarity between the training set and test set. All experiments in our manuscript follow these split datasets and show the results based on the average of the outputs from the models in the 5-fold cross-validation.

In addition to the ZDOCK benchmark 4.0 dataset, we also used benchmark 5.5 for the independent test set. The ZDock benchmark 5.5 includes 97 target complexes that are independent of the ZDOCK benchmark 4.0 dataset.

Three indicators iRMSD (root mean square deviation of the interface residues), fnat (the fraction of receptor-ligand residue contacts in the native structure that is reproduced in the decoy) and lRMSD (root mean square deviation of the ligand residues) (Gabb et al., 1997) were calculated for each docking model. According to the CAPRI quality assessment criteria, incorrect models were defined as follows:

Incorrect: fnat<0.1 or (lRMSD>10.0 and iRMSD>4.0) 

We took the protein docking models falling into this category as negative samples, and others as positive samples. However, some protein complexes in the ZDOCK decoy set did not contain any near-native docking models based on CAPRI criterion. Besides, lRMSD, fnat and iRMSD of some protein docking models could not be computed owing to the inconsistency of the sequences between decoys and native complexes (Wang et al., 2021). After filtering out these complexes, we obtained 144 usable protein complexes for our experiments. These protein complexes were divided into 5-fold cross-validation sets. To conduct ensemble learning, we bootstrapped 100 balanced training sub-sets, which included all positive samples and the same number of negative samples randomly extracted from all docking models of each protein complex. But we still evaluated our method on all 3,600 docking candidate models of each protein complex.

### Graph Construction

The 3-D structure of each candidate docking model was transformed to a graph, where its nodes were all residues at the model and its edges were defined according to the distance between any atoms of two residues. If the distance is less than 5 Å, we assigned an edge to connect them. After obtaining the graph structure of each protein docking model, we encoded each node (residue) with physico-chemical properties and sequence conservation information. For residue physico-chemical properties, we employed five principal components from 237 AAIndex physical-chemical properties presented by Mathura (Venkatarajan and Braun, 2001) to derive quantitative representations for each amino acid category. In addition, since the residues at the protein-protein interfaces are more conserved than other residues (Andreani and Guerois, 2014), we also calculated Position-Specific Scoring Matrix (PSSM) (Stormo et al., 1982) and the information content (IC) (Hennessey and Johnson, 1981) with 21-dimensional components as a part of residue (node) feature. Eventually, each residue (node) was encoded to a 26 × 1 vector by combining the 5-D physio-chemical properties and 21-D PSSM features of the residue profiles.

### Graph Neural Network Architecture

Since we formulated the protein docking model QA as a graph classification problem, any graph neural network for graph classification can be used as a classifier. Here, we modified DGCNN (Zhang et al., 2018) with GAT layers and named DGANN as shown in Figure 2. Our DGANN architecture included the GAT module, top-k-pooling module, and QA scoring module. The source code of DGANN is available at https://github.com/coffee19850519/PPDocking/tree/master.

**GAT module** stacks two GAT layers to learn node embeddings by aggregating their neighbors’ features under the style of self-attention. This type of layer can specify different weights to different neighboring nodes according to their node attributes. The attention coefficients $e_{ij}$ between node *i* and its first-order neighbor *j* are calculated as follows:

$$e_{ij}=W{\vec{h}}_{i}\times W{\vec{h}}_{j}$$

where *W* is a learned weight matrix, ${\vec{h}}_{i}$ and ${\vec{h}}_{j}$ represent embedding of node *i* and node *j*. $e_{ij}$ indicates the importance of node *j* to node *i*. And then $e_{ij}$ is normalized across all choices of j using a softmax function:

$$\alpha_{ij}=\text{soft}\max\left(e_{ij}\right)=\frac{\exp\left(e_{ij}\right)}{\sum_{k\in N_{i}}\exp\left(e_{ik}\right)}$$

Finally, the embedding of node *i* can be computed with an activation faction by the following equation:

$${\vec{h}}_{i}^{'}=\sigma \left(\sum_{j\in N_{i}}\alpha_{ij}W{\vec{h}}_{j}\right)$$

In our work, the activation function is tanh. Through these calculations, each 26-D input node encoding is mapped into the 32-D node features by using shared weights with the shape of 26 × 32. The node features implicitly include the interactions between two direct neighboring residues and local structural information. The upper GAT layer aggregates longer distanced neighbors’ node embeddings and maps them to a scalar by using another shared weights with the shape of 32 × 1 for each node, which is deemed as the importance of the node over the whole graph. We converted the entire docking model into a graph and learned embedding of all residues by stacking GAT layers over the whole graph.

**Top-k-pooling module** was utilized to obtain a fixed size of graph-level representations for different protein docking models. Since the protein-protein interface is the most distinctive and informative region, we only pooled the residue (node) embeddings at protein-protein interface to a graph-level representation. At this stage, only the interface residues are used in the pooling operations. However, because the number of residues at interfaces varied among protein docking models, we needed to unify the size of graph-level representations for further prediction. We used the top-k-pooling operation proposed by DGCNN to sort the node embeddings according to their importance and keep the top k nodes. For the models with fewer than 100 interface residues, we padded the placeholders 0 to fix the output shape of top-k-pooling. And then the two GAT outputs of the selected *k* nodes are read out and concatenated to form the graph-level embeddings of protein-protein interfaces.

**QA scoring module** supplied a 1-D convolutional layer and a fully connected layer to generate flatten feature vectors on the graph-level embeddings. Finally, a sigmoid function is employed to predict the probabilities of native-like models as QA scores.

### Deep Graph Attention Neural Network Training details

In the training stage, we adopted binary cross-entropy (Goodman, 2001) as a loss function. Adam optimizer (Kingma and Ba, 2014) with 0.001 learning rate and 0.9 decay were employed to train the network weights on mini-batches mode. The batch size was set to 256. We also applied the early stopping strategy to check if the loss of a validation set did not decrease within continuous 30 epochs. A dropout of 0.5 was also introduced to the fully connected layers. As mentioned in *Pipeline of Our Method Section*, we performed 5-fold cross-validation and trained our DGANN on 100 balanced bootstrapping subsets for ensemble learning. But for validating DGANN, all trained classifiers were tested on all imbalanced 3,600 candidate docking models of each protein complex.

### Evaluation Metrics

To validate the performance of our method, we used two evaluation measures: Enrichment Factor (EF) and success rate. The EFx% was defined as the ratio of the number of near-native models at x% top ranks relative to the number of testing docking models from a given protein-protein complex as follows:

$$EF_{\mathrm{x\%}}=\frac{\mathrm{positive}\;\mathrm{samples}\;\mathrm{at}\;\mathrm{x\%}}{\mathrm{all}\;\mathrm{samples}\;\mathrm{at}\;\mathrm{x\%}}\times \frac{\mathrm{total}\;\mathrm{samples}}{\mathrm{total}\;\mathrm{positive}\;\mathrm{samples}}$$

$$EF_{\max}\;=\;\frac{\mathrm{total}\;\mathrm{samples}}{\mathrm{total}\;\mathrm{positive}\;\mathrm{samples}}$$

where EF_max_ is the ratio of all docking models and native-like models, which indicates the degree of difficulty to select the positives from all docking models of a given protein-protein complex. EF shows the ability to enrich the number of native-like models at the top-scoring points compared to a random selection. Success rate is the percentage with at least one successful prediction when having a certain number of top ranks. In our experiments, each test protein complex’s metric values were averaged over outputs from the models in the 5-fold cross-validation as the final QA performance.

## Conclusion

In this study, we presented a deep graph neural network-based approach named DGANN for protein docking QA. We first formulated protein docking QA as a graph classification problem and converted 3D structures of protein docking models into graphs. In DGANN, we used GAT layers to aggregate the neighboring residue properties using the attention mechanism, which implicitly captured neighboring residue interactions and local topology information in node embeddings. Such multi-body interactions at residues were shown to be effective in selecting native-like protein structure models. Furthermore, a top-k-pooling strategy was employed to select a fixed size of residue (node) embeddings from protein docking interfaces to form graph-level representations for prediction. Our GNN model is significantly different from an earlier GNN model for docking evaluation (Wang et al., 2021). In the definition of our graph, we treated the residues at docking models as nodes instead of atoms in that work, which reduced the graph complexity and computational time cost. In addition, our graph is based on the whole protein model instead of interface only, which provided the full three-dimensional structures for accurate node embedding generation. The experimental results on the ZDOCK benchmark decoy set showed that our DGANN outperformed ZDOCK and classical deep learning approaches in terms of EF and success rate. In order to address the extremely imbalanced issues, we applied an ensemble strategy to integrate multiple classifiers over bootstrapping balanced training data. From experimental results, we can observe ensemble results were more stable and robust on imbalanced testing protein docking models.

Our work demonstrated that graph neural network can naturally extract the multi-residue interaction and topology information from molecular structures. For future work, we plan to include more residue features, such as secondary structural descriptors, solvent accessibility and electrostatic effects, into the node attributes for further improving graph representations. In addition, we will explore constructing atomic level graphs of protein docking models, which may provide higher-resolution predictions. And more advanced graph classification GNN architectures should be explored in the field of protein docking QA in the future. Moreover, our work also indicates the power of ensemble learning to address the imbalanced problem. However, only simple linear combination strategies were implemented in this work. Nonlinear ensemble i.e., stacking strategies (Breiman, 1996) may also contribute to revealing the potential of GNN in protein docking QA.

## Data Availability

Publicly available datasets were analyzed in this study. This data can be found here: https://zlab.umassmed.edu/benchmark/
